# Risk and protective factors of depression in the general population during the COVID-19 epidemic in Korea

**DOI:** 10.1186/s12888-021-03449-y

**Published:** 2021-09-08

**Authors:** Sung-Wan Kim, In-Hoo Park, Mina Kim, A-La Park, Min Jhon, Ju-Wan Kim, Hee-Ju Kang, Seunghyong Ryu, Ju-Yeon Lee, Jae-Min Kim

**Affiliations:** 1 Department of Psychiatry, Chonnam National University Medical School, 42 Jebong-ro, Dong-gu, Gwangju, 61469 Republic of Korea; 2 Gwangju Mental Health Commission, Gwangju, Korea; 3 Care Policy and Evaluation Centre, Department of Health Policy, London School of Economics and Political Science, London, United Kingdom

**Keywords:** Depression, COVID-19, gratitude, loneliness, stress, exercise

## Abstract

**Background:**

The risk of depression has risen in the general population during the COVID-19 epidemic. This study was conducted to explore risk and protective factors associated with depression among the general population uninfected by COVID-19.

**Methods:**

A cross-sectional study was conducted with 1,500 representative South Korean citizens aged 19–65 years through an anonymous online survey. Depression was defined as a Patient Health Questionnaire-9 score of 10 or higher. Other questionnaires included one measuring psycho-behavioural and social changes, and stress, due to COVID-19, a six-item version of the Gratitude Questionnaire (GQ-6), and a three-item version of the UCLA loneliness scale.

**Results:**

Of the 1492 participants not infected by COVID-19, 312 (20.9%) exhibited depression. Multiple logistic regression analysis revealed that depression was positively associated with COVID-19-related stress and psycho-behavioural variables such as disturbances in eating and sleeping, younger age, smoking, underlying mental illness, and loneliness scale scores. In contrast, exercise three or more times per week and GQ-6 scale scores were inversely associated with depression.

**Conclusion:**

During the COVID-19 pandemic, maintaining daily routines including eating, sleeping, and regular exercise and focusing on gratitude may be important for the prevention of depression. In addition, more attention should be paid to vulnerable populations, including young people, those with mental illnesses, and smokers, who might be more susceptible to depression.

## Background

The Corona Virus Disease 2019 (COVID-19) pandemic has drastically changed our society and infiltrated every aspect of our daily lives [1]. Long-lasting social distancing and shrinkage of economic activity tend to lead to depression [2]. The risk of depression has risen in the general population as well as in those with COVID-19 infection [3, 4]. Most people, regardless of their viral infection status, have been affected by the psychosocial changes associated with the COVID-19 pandemic [5]. Depression adversely affects individuals’ mental and physical health and the social and financial welfare of the individual and society [6]. Thus, the higher prevalence of depression during this pandemic may be a crucial component of the increased socioeconomic burden imposed by COVID-19.

The causes of depressive disorder are generally heterogeneous. Various psychosocial factors, many associated with the pandemic, are likely related to the increased risk for depression: female gender, unmarried status, lower income, and negative economic impacts [3, 6, 7]. Underlying physical and psychiatric illness, emotional stress, and loneliness are also associated with increased risk for depression [6, 8]. In addition, lifestyle disturbances such as changes in sleep and nutrition, can increase depression risk [9].

In contrast, active coping strategies with positive emotions and social support have been reported as protective factors [10]. Positive psychological factors, including optimism, altruism, and gratitude, may reduce perceived stress and heighten the sense of well-being [11, 12]. In a natural disaster or pandemic, altruistic acceptance can help prevent negative impacts on mental health and depression [13]. Gratitude is associated with positive reframing and emotions that are, in turn, associated with fewer symptoms of depression [11, 14, 15]. Gratitude has also been reported to protect against depressive symptoms following traumatic events [16]. In our previous investigation of the association of gratitude with perceived stress and burnout among male firefighters, gratitude independently protected against stress and burnout [17]. Because the COVID-19 pandemic is a community disaster with many unwanted social restrictions, we hypothesised that gratitude might be important in protecting against depression. Although gratitude has been reported to have a positive impact on the mental health of service providers [18], few studies have investigated its protective effects on depression in the general population during the COVID-19 pandemic. Regular physical exercise can also provide protection against future depression [19]. However, restrictions on activities and social interactions during the COVID-19 pandemic have exacerbated already-stressful socioenvironmental conditions, enhancing risk factors and hindering protective factors against depression.

Insufficient evidence has been available regarding various psychosocial factors associated with depression among the general population in COVID-19-impacted societies. This study investigated the risk and protective factors associated with depression in the general population uninfected by COVID-19. In Korea, after the first patient with COVID-19 was identified on 20 January 2020, the number of confirmed patients began to increase sharply from mid-February. The number of confirmed cases in Korea was ranked second highest in the world at that time. The Korean Government declared a pandemic in late February. Although Korea did not undergo lockdown, the start of the school year was postponed, most social events were cancelled, and the general population was asked to continue social distancing. By the end of April, when this survey began, the number of confirmed cases exceeded 10,000 nationwide (ranked 30^th^ highest worldwide), and the first wave of the COVID-19 outbreak, throughout which social distancing had been maintained, had passed.

Our hypothesis was as follows. Poor socioeconomic status and COVID-19-related stress caused by long-lasting social distancing may be associated with an increased risk for depression. In addition, despite the poor social environment associated with the COVID-19 pandemic, maintaining a positive mindset, a daily routine, and physical activity may be protective against depression. For these purposes, we analysed socioenvironmental variables, distress, and psychosocial experiences associated with COVID-19, the frequency of physical exercise, and psychological scales examining loneliness and gratitude. We believe that our study could contribute to the development of a basic coping strategy during this pandemic, based on risk and protective factors for depression.

## Methods

### Study design and participants

This was an online-based cross-sectional study that was part of a survey on the psychosocial effects of COVID-19 in the general population. Details of the data collection method were described previously [20]. Briefly, between April 24 and May 5 of 2020, the third month of the COVID-19 outbreak in South Korea, we conducted an anonymous online survey of residents aged 19–65 years from three metropolitan areas according to prevalence of COVID-19. The online survey was conducted via a service provider (Macromill Embrain). For this survey, an invitation e-mail was sent to 4,065 individuals registered in the survey pools, of whom 3,065 (75.4%) accessed the link to the survey instructions. Of these, 1,206 (39.3%) were excluded because they did not meet the survey criteria and 40 (1.3%) refused to take part in the survey. A total of 1,819 people completed the survey and data for 1,500 were finally collected, after excluding 319 (17.5%) with unreliable or invalid responses including the same or a stereotyped pattern of responses to questions and illogical responses. In this study, data from 1,492 respondents with no history of COVID-19 infection (excluding eight confirmed cases of COVID-19) were used. The study was approved by the Chonnam National University Hospital Institutional Review Board (CNUH-2020-092). Electronic informed consent was obtained from each participant prior to starting the investigation.

### Outcome measures

Demographic variables included sex, age, marital status (married or not), residential location, religious identity, education level, medical insurance (Medicaid or Medicare), employment status (unemployed or temporarily employed vs. employed, student, or homemaker), frequency of exercise (three times or more per week before and during the COVID-19 pandemic, respectively), and smoking status. Clinical characteristics included underlying physical or mental illness (defined as currently taking medication for a condition), quarantine experience, and COVID-19 testing.

Depression was measured using the Patient Health Questionnaire-9 (PHQ-9) [21]. A cut-off score of ≥ 10, was used as an indicator of clinically relevant symptoms of depression according to a previous study showing 88% sensitivity and specificity [22]. Gratitude and loneliness as indices of psychological status were measured using a six-item version of the Gratitude Questionnaire (GQ-6) [23] and the three-item UCLA Loneliness Scale [24, 25]. The Korean versions of these scales were validated with favourable reliability and validity [26–28]. Cronbach’s alpha values of all these measures are above 0.8 in the present study.

A questionnaire developed and validated by the authors was used to identify psychological and behavioural changes and distress due to the spread of COVID-19. The psychometric properties of this questionnaire are presented in a previous paper [20]. All items in the questionnaire were rated using a five-point Likert scale. The COVID-19-related questionnaire included five factors: fear of COVID-19 infection (seven items), difficulties related to outside activities (four items), disturbances in eating and sleeping (three items), stigma of COVID-19 (three items), and fear of blame for COVID-19 infection (two items). In addition, six items related to stress associated with the COVID-19 pandemic, including economic stress and difficulty with obtaining daily necessities, were included. Scores on all subfactors of the questionnaire are presented as mean values (total scores divided by number of items).

### Statistical analysis

Differences in sociodemographic, clinical, and psychosocial characteristics for those with depression measured by PHQ-9 were analysed using the chi-square test for categorical variables and independent t-tests for continuous variables. Variables that were significantly associated with depression at the *p <* 0.05 level in the univariate analyses were simultaneously entered into a multiple logistic regression analysis to identify factors independently associated with depression. SPSS for Windows software was used to perform the statistical tests. All statistical tests were two-tailed, and *p*-values <0.05 were considered significant.

## Results

Among 1,500 members of the general population, eight people (0.5%) had been infected by COVID-19. The prevalence of depression, indicated by PHQ-9 scores≥10, was 21.1% in the total population (316 of 1,500 people) and 20.9% without COVID-19 (312 of 1,492). Half of infected people (four of eight) exhibited depression, but this incidence was not significant (*p* = 0.066).

Table 1 shows comparisons of sociodemographic factors and scores on clinical scales with or without depression in the general population uninfected by COVID-19. Unmarried people, smokers, people on psychiatric medication, and unemployed or temporarily employed people were significantly more likely to exhibit depression. Those with depression were significantly younger than those without. In contrast, people who were currently exercising at least three times weekly were significantly less likely to exhibit depression. However, the frequency of exercise before the pandemic was not significantly associated with depression. There was no significant difference in depression according to sex, religion, region, or presence of a physical illness treated by medication.

**Table 1:** Comparisons of sociodemographic factors and clinical scales according to the presence of depression

	Total	Depression (+)	Depression (-)	Statistics	***P-***value
	(N=1492)	(N=312, 20.9%)	(N=1180, 79.1%)		
Sex, n (%), Female	748 (50.1)	158 (50.6)	590 (50.0)	χ^2^ = 0.041	.840
Age, mean (SD), years	40.3 (11.8)	37.5 (11.2)	41.0 (11.9)	t = 4.654	**<.001**
Region, n (%)					
Capital area	500 (33.5)	112 (35.9)	388 (32.9)	χ^2^ = 3.257	.196
Daegu	493 (33.0)	109 (34.9)	384 (32.5)		
Gwangju	499 (33.4)	91 (29.2)	408 (34.6)		
Marital status, n (%), Unmarried	755 (50.6)	199 (63.8)	556 (47.1)	χ^2^ = 27.411	**<.001**
Education, n (%), High school graduate/dropout or less	255 (17.1)	62 (19.9)	193 (16.4)	χ^2^ = 2.153	.142
Occupational type, n (%), Unemployed or temporary workers	305 (20.4)	93 (29.8)	212 (18.0)	χ^2^ = 21.275	**<.001**
Medical security, n (%), Medicaid	69 (4.6)	20 (6.4)	49 (4.2)	χ^2^ = 2.852	.091
Religious status, n (%), yes	580 (38.9)	110 (35.3)	470 (39.8)	χ^2^ = 2.173	.140
Smoking status, n (%), yes	318 (21.3)	88 (28.2)	230 (19.5)	χ^2^ = 11.171	**.001**
Underlying mental illness, n (%), yes	67 (4.5)	27 (8.7)	40 (3.4)	χ^2^ = 15.942	**<.001**
Underlying physical illness, n (%), yes	398 (26.7)	77 (24.7)	321 (27.2)	χ^2^ = 0.804	.370
Quarantine experience, n (%), yes	33 (2.2)	11 (3.5)	22 (1.9)	χ^2^ = 3.148	.076
Frequency of exercise					
Three times or more per week before COVID-19 pandemic, n (%)	891 (59.7)	191 (61.2)	700 (59.3)	χ^2^ = 0.369	.544
Three times or more per week during COVID-19 pandemic, n (%)	464 (31.1)	71 (22.8)	393 (33.3)	χ^2^ = 12.814	**<.001**
COVID-19-related questionnaire					
Stress associated with the COVID-19 pandemic	18.2 (4.6)	20.2 (4.5)	17.7 (4.5)	t = -8.754	**<.001**
Fear of COVID-19 infection	3.8 (0.7)	3.9 (0.8)	3.8 (0.7)	t = -2.764	**.006**
Difficulty in outside activities	4.2 (0.6)	4.3 (0.6)	4.2 (0.6)	t = -0.619	.536
Disturbance in eating and sleeping	2.8 (0.9)	3.3 (0.9)	2.7 (0.9)	t = -9.845	**<.001**
Stigma of COVID-19	2.9 (0.8)	3.0 (0.8)	2.8 (0.8)	t = -4.383	**<.001**
Fear of blame for COVID-19 infection	3.6 (1.0)	3.7 (0.1)	3.5 (1.0)	t =- 3.049	**.002**
Psychiatric scales					
UCLA loneliness scale-3	5.2 (1.7)	6.7 (1.7)	4.8 (1.5)	t =-17.959	**<.001**
Gratitude questionnaire-6	30.2 (6.1)	26.9 (6.2)	31.0 (5.7)	t = 10.970	**<.001**

Bold values denote statistical significance at the *p* < 0.05 level.

The loneliness scale scores were significantly higher, and the GQ-6 scores significantly lower, in people with depression than in those without. Of the items used to assess COVID-19 distress, scores for fear of infection, disturbance in eating and sleeping, stigma of COVID-19, fear of blame for COVID-19 infection, and stress associated with COVID-19 were significantly higher in people with depression.

Table 2 shows the results of the logistic regression analysis of factors associated with depression. Higher scores on the loneliness scale, younger age, current smoking, and current psychiatric treatment were significantly associated with increased risks of depression. Of the questions specifically asking about COVID-19-related distress, disturbances in eating and sleeping and the level of stress due to COVID-19 were significantly associated with increased risks of depression. In contrast, exercising three times or more per week during the COVID-19 pandemic and scores on the GQ-6 were significantly inversely associated with depression. The proportion of the explained variance (Nagelkerke R^2^) in this logistic regression model was 36.4%.

**Table 2:** Multiple logistic regression analysis to detect factors associated with depression

Variable	Wald	OR	95% CI	***P-***value
Age; year	4.294	0.983	0.967-0.999	**.038**
Marital status, unmarried	1.201	1.241	0.843-1.828	.273
Occupational type, unemployed or temporary workers	0.291	1.104	0.771-1.580	.589
Smoking status, yes	4.319	1.439	1.021-2.028	**.038**
Underlying mental illness, yes	4.363	1.950	1.042-3.648	**.037**
Exercise, three times or more per week during COVID-19 pandemic	5.171	0.672	0.477-0.947	**.023**
Stress associated with the COVID-19 pandemic; score	11.442	1.077	1.032-1.125	**.001**
Fear of COVID-19 infection; score	3.335	0.781	0.599-1.018	.068
Disturbance in eating and sleeping; score	21.072	1.558	1.289-1.883	**<.001**
Stigma of COVID-19; score	0.962	1.105	0.905-1.349	.327
Fear of blame for COVID-19; score	0.005	0.994	0.831-1.188	.945
UCLA loneliness scale-3; score	110.950	1.646	1.500-1.806	**<.001**
Gratitude questionnaire-6; score	27.295	0.933	0.908-0.957	**<.001**

Nagelkerke *R*^2^=0.364; Bold values denote statistical significance at the *p* < 0.05 level.

*Abbreviations*: *CI* confidence interval, *OR* odds ratio

## Discussion

Our study results showed that during the COVID-19 pandemic, about one in five members of the general population who were not infected had clinically significant depression. Thus, the prevalence of depression was rather high. The risk of depression was increased in people suffering from loneliness and COVID-19-related stress, people who experienced psycho-behavioural changes such as disturbances in eating and sleeping, smokers, people with underlying mental illness, and those who were younger. However, regular exercise and attitudes of gratitude were inversely associated with depression. Our study demonstrated that psychosocial changes and COVID-19-related stress increased the risk of depression and suggested that maintaining life routines such as regular exercise, eating, and sleeping and positive mind may be helpful in preventing depression during these COVID-19 times.

The prevalence of depression (21%) found in this study was similar to that of previous reports about depression prevalence (PHQ-9≥10) during the COVID-19 pandemic in the US, Europe, and Asian countries (14.4–27.8%) [2, 4, 29–31]. According to the 2019 Korea Community Health Survey, the prevalence of depression in Korea, using the PHQ-9, was only 4.3% in 2018 [32]. Although there are limitations to comparing subjects and methods directly across studies, our results, showing an approximately fivefold increase in the prevalence of depression, suggest that psychosocial changes induced by the COVID-19 pandemic increased the risk of depression. The development of psychosocial strategies for the prevention of depression is urgently needed. For this, we should first understand the pathogenesis of, and factors associated with depression in COVID-19 pandemic societies.

In this study, the stress associated with psychosocial changes due to COVID-19 was significantly associated with increased risks of depression. In previous studies, groups with high COVID-19-related stress had an increased risk of depression compared to those that did not [33]. When people are exposed to situations beyond their control, they become helpless and lack motivation, resulting in depression [34]. The societal changes caused by the rapidly increasing number of confirmed COVID 19 cases and deaths have exacerbated uncertainty and reduced individuals’ sense of control. Furthermore, restriction of social and economic activities may increase the risk of cause depression.

Disturbances of sleep and eating patterns related to COVID-19, which were also significantly associated with increased risks of depression, might be more easily modified at a personal level. While a causal relationship is unclear, the inverse relationship between regular exercise during the pandemic and depression suggests that maintaining healthy lifestyles like regular exercise, eating habits, and sleep patterns daily may be important tools for preventing depression in the COVID-19 era [35] by improving mental and physical health. Our findings are consistent with a Spanish study showing that a healthy, balanced diet, following daily routines, and maintaining daily outdoor activities were coping behaviours that could reduce depression [36].

Loneliness was strongly associated with the risk of depression in our study. Loneliness is also associated with social isolation and appears to mediate depression [37]. Although social distancing is effective in preventing the coronavirus spread, it can cause loneliness due to emotional disconnection [30]. Despite long-lasting and inevitable social distancing policies, social and emotional interactions may reduce impacts of loneliness on depression.

In recent studies, positive traits such as optimism and resilience significantly moderated the relationship between fear of COVID-19 and depression and reduced the negative effects of COVID-19 on mental health [38]. In this study, gratitude was inversely associated with depression, corroborating findings that gratitude as a form of resilience has a buffering effect on the negative mental health consequences of stressful events [39]. Gratitude is an expression of appreciation for people or things in one’s life [40]. It is associated with several positive traits, including social support, self-esteem, and satisfaction with life [23]. At societal levels, gratitude is also important in forming and maintaining social relationships and encouraging people to be more prosocial [41]. Our results suggest that, during this COVID-19 pandemic, people are experiencing social isolation and loneliness, gratitude can be a psychological antidote to depression by reinforcing social bonds as well as positive mood. Although the COVID-19 pandemic and associated societal changes are drastically influencing our lives, positive attitudes and a sense of gratitude are likely to be helpful in preventing depression. Gratitude can be cultivated and trained by personal practice or group programs (e.g. writing a gratitude diary, expressing gratitude in a group or within couples) [42, 43]. Therefore, a public campaign to express gratitude and applying gratitude interventions in clinical practice, can be beneficial for coping with stress during the COVID-19 pandemic.

Socially and mentally vulnerable populations, such as the unemployed or temporary workers as well as people with underlying psychiatric illnesses, were more likely to have current significant depressive symptoms, although employment status was no longer significant in the adjusted analysis. Mentally ill persons may have limited social and medical support after the COVID-19 pandemic. Therefore, it is necessary to provide continuing care and carefully observe those with underlying psychiatric illnesses. In other studies, concerns about losing jobs and unemployment status increased depression [36, 44]. As COVID-19 transforms the labour market, individuals with precarious employment may show increased susceptibility to depression [45]. We should pay more attention to socially and mentally vulnerable people exposed to social and natural disasters.

The younger generation tended to be at greater risks of depression. Generally, the prevalence of depression is higher in middle aged and elderly people [46]. However, many recent studies have found that young people were more vulnerable to mental health problems during the COVID-19 pandemic era [3, 21, 30, 31, 47–49]. Current drastic social changes may have greater impacts on the younger generation, whose psychological resilience and established social base may be inadequate for this situation. They may experience greater fear and worry about their future while living in this socioeconomically unstable society. In addition, young people are at greater risks for emerging psychiatric problems. Taken together, these results indicate that populations who are psychosocially vulnerable may be more severely affected by the distress associated with COVID-19. Therefore, we should observe these vulnerable populations carefully to facilitate early detection of mental health problems and to provide more active support [50].

Cigarette smoking is reportedly associated with increased risks of depression [51]. Recent studies investigating factors associated with depression also showed that smoking was associated with depression during the COVID-19 pandemic [52]. Both the biological impacts of smoking on neurotransmitters and vascular health [53] and the psychosocial influence of this respiratory pandemic on smokers should be considered as factors in the pathogenesis of depression in smokers.

This study has several limitations. First, we should be careful in applying our results to other populations affected by COVID-19. Second, causal relationships are uncertain because this study was cross-sectional. In particular, disturbances in eating and sleeping are symptoms of depression. Longitudinal studies are needed to investigate the risks and protective factors of depression associated with the COVID-19 pandemic. Finally, results via an online survey method may differ from those acquired through face-to-face surveys, and there is a potential for selection bias. Therefore, caution is required when comparing the results of online and face-to-face surveys.

## Conclusions

Findings of the factors associated with increased risks of depression in the general population during the COVID-19 pandemic offer important clinical implications for the general population as well as for researchers. We should pay more attention to the younger people, smokers, and people with mental health problems who might be at higher risks of depression. Psychological support for the loneliness and distress due to psychosocial changes related to COVID-19 are required to prevent depression. Furthermore, maintaining daily routines, including a regular/balanced diet, sleeping, and exercise, and having a sense of gratitude may be helpful in preventing depression. Although it has been quite difficult to overcome the COVID-19 pandemic and its associated drastic societal changes, adhering to daily routines with gratitude in mind is a relatively straightforward way to prevent depression. These coping behaviours can be recommended to the public during this exceptional situation, and should be encouraged, even with social distancing measures in place [36]. Additional studies investigating the effectiveness of psychosocial strategies to prevent depression should be conducted.

## Data Availability

The dataset supporting the conclusions of this article is available on request from the first author.
